# The prevalence and stressors of job burnout among medical staff in Liaoning, China: a cross-section study

**DOI:** 10.1186/s12889-021-10535-z

**Published:** 2021-04-23

**Authors:** Youqi Guo, Shu Hu, Fei Liang

**Affiliations:** 1 College of the Humanities and Social Sciences, China Medical University, Shenyang, People’s Republic of China; 2 Department of Histology and Embryology, College of Basic Medicine, China Medical University, Shenyang, People’s Republic of China

**Keywords:** Burnout, Chinese medical staff, Stressors, Relationship with patients, Work tasks, Workplace violence, Fear of malpractice

## Abstract

**Background:**

Sustained attention to the prevalence and associated factors of burnout in China is important for the health care service quality and related reform. In this study, we investigated the prevalence of job burnout among medical staff in Liaoning province, China; performed a survey of subjective perception ranking for the main stressors among respondents; estimated the effect of stresses from work tasks and the relationship with patients on job burnout in order to provide improved strategy and suggestion for hospital administrators.

**Methods:**

The respondents were from 8 hospitals in 3 cities in Liaoning province, China. Data were collected and analyzed including the following sections: (1) demographic characteristics; (2) work situations; (3) ranking of six stressors; (4) job burnout scale; (5) effort-reward imbalance scale; (6) work violence scale; (7) fear of malpractice scale. A total of 1056 individuals became the study objects. A statistical analysis and hierarchical linear regression analysis were performed to explore the prevalence of burnout and the effects of stressors.

**Results:**

The prevalence of job burnout was 20.5, and 72.9% of all respondents reported a least one symptom of burnout. The respondents who were male, 30–39 years old, had a master’s degree or high and working hours > 60 h per week, came from obstetrics and gynecology or pediatrics profession prone to job burnout. The relationship with patients and work tasks are the top two ranking stressors in the subjective perception survey. Regression analysis showed that the relationship with patients explained 19.2, 16.8 and 2.0% of variance in burnout subscales EE, DP and PA, respectively and work tasks explained 23.5, 16.0 and 5.24% of variance in burnout subscales EE, DP and PA, respectively.

**Conclusion:**

The Chinese medical staff had high prevalence of job burnout. Some factors of demographic and work situations were associated with job burnout. The medical staff considered the relationship with patients and work tasks are the two major stressors. These two stressors are also the major indicators associated with job burnout. The hospital administrators should be aware of the risk of burnout. Efforts should be made to ameliorate the status of job burnout.

## Background

Burnout is a psychological syndrome described as “emotional exhaustion and cynicism that occurs frequently among individuals who do people-work” [1]. The theoretical framework of burnout generally has 3 key dimensions: emotional exhaustion (EE), depersonalization (DP) or cynicism, and reduced or lower personal accomplishment (PA) or efficacy [2]. Burnout is a common experience for doctors and nurses. The aggregate global prevalence rate of burnout among residents was 51.0% [3]. A burnout review showed the overall prevalence of physicians in different definitions and criteria ranged from 0 to 80.5%, EE 0 to 86.2%, DP 0 to 89.9%, and low PA 0 to 87.1% [4]. Another systematic review showed the global prevalence of burnout symptoms among nurses was 11.23% [5]. In China, medical staff are also considered as a risk population with burnout [6, 7]. A systematic review showed the rate of burnout among doctors in China was between 66.5 to 87.8% [8]. A national survey for Chinese nurse reported 50% of the participants suffered burnout [9]. As the outcome, job burnout was linked with poor health and job withdrawal such as increased absenteeism and turnover rate [10–13]. Burnout among medical staff has become a public health crisis that needs urgent action [14]. In China, due to the increasing demand from the vast population and limited available medical resources, including human resources, medical staff frequently experience work-related stress and energy deficiencies, which may deteriorate the situation of job burnout [15, 16]. Sustained attention to the prevalence and associated factors of burnout in Chinese medical staff is important to increase the health care service quality and related reform.

The relationship between the three dimensions of burnout was often described in a sequential conceptual model: EE, DP will sequentially occur in response to job demands and overload, then reduced PA will simultaneously or sequentially develop [2, 10]. However, the stress and burnout have a causal relationship [17], job stressors play an important role for burnout. Another burnout conceptual model has three stages: job stressors, individual strain (emotion response), and defensive coping (attitudes and behavior changes, such as cynicism) [10]. These models highlight the importance of job stressors. The general notion is that burnout is the end result of long exposure to chronic interpersonal stressors on the job [10, 18]. Upon this notion, we considered that the main stress among Chinese medical staff come from their work itself and the job-related interpersonal relationship. Among Chinese medical staff, the former stressors mainly refer to such as work tasks and title promotion, the latter refers to such as the relationship with patients, superiors, colleagues and family.

The stress from work tasks refers to the perceived stress from routine medical job such as job demands and workload, uncertainty concerning treatment etc., which is the main stressor for work itself. The work stress is the importance factor for burnout [2, 19]. Among the various theoretical models for work stress, the Job Demand-Control (JDC) model [20] and the Effort-Reward Imbalance (ERI) model [21] were often used. The Job Content Questionnaire (JCQ) is based on JDC model, focusing on extrinsic workplace atmosphere and job characteristic, while the ERI scale concentrates more on intrinsic individual cognition of the imbalance of pain and gain [22]. In this study, we used ERI to evaluate perceived stress from work tasks. In China, the title promotion is mainly based on academic performance such as publishing SCI paper and fund application etc. However, these works were not usually included in their daily job content, and it was generally regarded as an independent stressor different from work tasks. The title rank was often discussed as an occupational factor in burnout studies [13, 15, 23], however, to our knowledge, there is no reliable separate scale to measure the stress from title promotion.

Among the job-related interpersonal relationships, the crisis of the relationship with patients was often mentioned in Chinese medical staff burnout study [24–27]. Tension relationship between patients and medical staff cause popular dissatisfaction and mistrust between them. Due to the dissatisfaction of patient, Chinese medical staff suffered with high prevalence of workplace violence [28]. A meta-analysis showed that the overall prevalence of workplace violence for Chinese health-care professionals was 62.4% [29]. In the other side, distrust from patients also causes popular defensive medical practice due to fear of malpractice [30]. Although there are some scales like the Patient–Doctor Relationship Questionnaire [31], the measurements for the relationship with patients are not generic. By combining workplace violence and fear of malpractice in one study, it can be objective measurement to evaluate the perceived stress from the relationship with patient among Chinese medical staff.

The other job-related interpersonal relationships like the relationships with superiors or colleagues, work-family conflict (WFC) have been found to be associated with burnout among Chinese medical staff [15, 19, 22, 32–35]. The relationships with superiors and colleagues were generally estimated by the social support subscale of JCQ [15, 22]. Work-family conflict (WFC) was usually measured by two subscales WIF (work interfering family conflict) scale and FIW (family interfering work conflict) scale. These stressors have been explored in previous burnout studies, however, these studies usually only discussed one or two certain stressors, lacking of the overall subjective perception survey among medical staff.

In this study, we designed a cross-sectional study to investigate the prevalence of job burnout among medical staff in Liaoning province, China. We also performed a subjective perception ranking survey for the main stressors among respondents. Meanwhile, we evaluated the effects of stresses from work tasks and relationship with patients on job burnout. Our study try to find effective ways of reducing burnout and provide improved suggestion for hospital administrators.

## Methods

### Study design and participants

A cross-sectional study was conducted in Liaoning province of China during September 2017 to January 2018. The doctors and nurses from 8 tertiary hospitals in 3 cities (Dalian, Shenyang and Chaoyang) were included in this study. A self-administered questionnaire was distributed among these medical staff. Eventually, we obtain 1070 returned questionnaires. However, 34 questionnaires were excluded because of missing demographic information (gender, age, marital and education). At last, 1056 questionnaires became the study objects.

The questionnaire used for this study includes following sections: demographic information (gender, age, marital and education), work conditions (position, professional title, income per month, working hours, professional department), work status (outpatient and inpatient practice), subjective ranking of six stressors, job burnout scale, effort-reward imbalance (ERI) scale, workplace violence scale (WVS) and fear of malpractice scale (FMS).

### Measurements

Ranking of six stressors were investigated among the participants. These six stressors include work tasks, relationship with patients, title promotion, relationship with superiors, relationship with colleagues and family conflict. The scores of six stressors were capsulated by the following formula:

$$ \mathsf{score}=\left(\mathsf{thenumberofranking}\mathsf{1}\right)\mathsf{X}\mathsf{6}+\left(\mathsf{thenumberofranking}\mathsf{2}\right)\mathsf{X}\mathsf{5}+\left(\mathsf{thenumberofranking}\mathsf{3}\right)\mathsf{X}\mathsf{4}+\left(\mathsf{thenumberofranking}\mathsf{4}\right)\mathsf{X}\mathsf{3}+\left(\mathsf{thenumberofranking}\mathsf{5}\right)\mathsf{X}\mathsf{2}+\left(\mathsf{thenumberofranking}\mathsf{6}\right)\mathsf{X}\mathsf{1} $$

#### Burnout

To evaluate job burnout, we used a standardized Chinese version of the Maslach Burnout Inventory-Human Service Survey (MBI-HSS). MBI-HSS consists of 22 items on a 7-point Likert-type scale ranging from 0 (never) to 6 (every day). MBI-HSS contains three subscales: emotional exhaustion (EE, 9 items), depersonalization (DP, 5 items), and personal accomplishment (PA, 8 items). Higher levels of burnout were positively associated with higher scores on EE and DP, and with lower scores on PA.

For each MBI-HSS subscale, the scores are categorized as low level (EE = 0–16, DP = 0–6, PD ≥ 39), moderate level (EE = 17–26, DP = 7–12, PA = 32–38), and high level (EE ≥ 27, DP ≥ 13, PA ≤ 31) [26, 36, 37]. The job burnout level is categorized as without burnout (without high level in all 3 subscales), low burnout (high in any one subscale), moderate burnout (high in two subscales), or high burnout (high in all three subscales). In this study, the Cronbach’s alpha coefficients for the MBI-HSS, EE, DP, and PA were 0.836, 0.878, 0.787, and 0.867 respectively.

#### ERI

Stress from work tasks was measured using the Chinese version of the Effort-Reward Imbalance (ERI) Questionnaire. The ERI consists of 23 items including three subscales: extrinsic effort (6 items), reward (11 items), and overcommitment (6 items) [15, 21, 22]. Extrinsic effort and reward were scored from 1 to 5; higher scores indicated higher demands of effort and rewards. Overcommitment was scored from 1 (complete disagreement) to 4 (complete agreement). The Cronbach’s alpha coefficients for extrinsic effort, reward, and overcommitment were 0.897, 0.910, 0.787, respectively.

#### WVS

We evaluate work violence by a Chinese version of the Workplace Violence Scale (WVS) consisting of 5 items on a 4-point Likert scale ranging from 0 (never) to 3 (more than 3 times/year) [25]. WVS included physical violence (physical aggression and sexual aggression) and nonphysical violence (verbal abuse, threats of violence, sexual harassment). In this study, the Cronbach’s alpha coefficient for the WVS is 0.812.

#### FMS

The Fear of malpractice scale (FMS) [38] includes 6 items on a 5-point Likert scale ranging from 1 (strongly disagree) to 5 (strongly agree). The sum of responses was calculated. Higher scores corresponding to increased malpractice fear. In this study, the Cronbach’s alpha coefficient for the FMS is 0.839.

### Statistical analysis

Student’s *t* test, an one-way analysis of variance (ANOVA) and the Kruskal-Wallis nonparametric test (in the occasion of uneven variance with ANOVA) were performed to examine the difference in MBI-HSS subscales among groups. Dependent variables (EE, DP and PA) were treated as continuous variables. Correlation between MBI-HSS subscales scores and ERI, WVS and FMS were examined by Pearson correlation. We performed a hierarchical linear regression analysis for each burnout subscale (EE, DP and PA). Variables including demographics (age, gender, education, and marital status), work situations (professional title, income per month, working hours), relationship with patient (including FMS and WVS) and work stress (ERI) were enter in the model.

All data from questionnaires were input in EpiData 3.0 software. Statistical analysis was performed by SPSS version 25.0 (IBM, Armonk, NY). All statistical tests were two-tailed, and *p* < 0.05 was significant.

## Results

The demographic and work condition characteristics of respondents in the survey were shown in Table 1. In this study, most of the respondents were female (71.2%), most respondents were less than 40 years old (45.7% were 30–39 years old and 24.3% were younger than 30 years old), and 70.9% respondents were married. About half of the respondents had a bachelor degree (50.3%), a quarter of the respondents had a master’s degree or higher (25.9%). 60.4% were doctors and 39.4% were nurses. Nearly half of the respondents had a junior professional title, the remaining had a middle or senior professional title. Most respondents reported their income per month is among 3000–8000 CNY (about 450 to 1200 dollars). Among all the respondents, 45.9% worked 40–49 h per week, 22.3% worked 50–60 h per week, 19.2% worked > 60 h per week. Most of the respondents came from internal medicine or surgery or surgical related department, 10.6% of respondents came from obstetrics and gynecology or pediatrics, 8.8% came from some small specialist like ophthalmology and otorhinolaryngology, dermatology or physical examination, and 15.1% came from auxiliary clinical or others department.

**Table 1:** Personal characteristics of the survey

Factors	
**Demographic**	*N* = 1056 (%)
**Gender**	
**Male**	304 (28.8%)
**Female**	752 (71.2%)
**Age(y)**	
**< 30**	257 (24.3%)
**30–39**	483 (45.7%)
**40–49**	205 (19.4%)
**≥ 50**	111 (10.6%)
**Marital status**	
**Single**	291 (27.6%)
**Married**	749 (70.9%)
**Divorced or widowed**	16 (1.5%)
**Education**	
**College degree or lower**	251 (23.8%)
**Bachelor degree**	531 (50.3%)
**Master ‘s degree or higher**	274 (25.9%)
**Work conditions**	*N* = 1056 (%)
**Position**	
**Doctor**	638 (60.4%)
**Nurse**	416 (39.4%)
**Missing data**	2 (0.2%)
**Professional Title**	
**Junior**	499 (47.3%)
**Middle**	363 (34.4%)
**Senior**	191 (18.1%)
**Missing data**	3 (0.3%)
**Income per month (CNY)**	
**< 3000**	223 (21.1%)
**3000-4999**	451 (42.7%)
**5000-7999**	328 (31.1%)
**> 8000**	52 (4.9%)
**Missing data**	2 (0.2%)
**Working Hours Per Week (h)**	
**< 40**	125 (11.8%)
**40–49**	485 (45.9%)
**50–60**	236 (22.3%)
**> 60**	203 (19.2%)
**Missing data**	7 (0.7%)
**Professional department**	
**Internal Medicine**	346 (32.8%)
**Surgery/ Emergency/ ICU/Anesthesia or operating room**	346 (32.8%)
**Obstetrics and Gynecology/Pediatrics**	112 (10.6%)
**Ophthalmology and Otorhinolaryngology/Dermatology/Physical examination**	93 (8.8%)
**Auxiliary Clinical (Laboratory, Ultrasound, Pharmacy, Radiology)/others**	159 (15.1%)
**Work status**	
**Outpatient practice**	*N* = 363 (%)
**Outpatient practice per week**	SD = 2.65
**1–2 times**	228 (62.8%)
**3–4 times**	61 (16.8%)
**≥ 5 times**	74 (20.4%)
**Clinic patients per day**	SD = 28.8
**< 10**	91 (25.1%)
**10 ~ 30**	162 (44.6%)
**30 ~ 50**	69 (19.0%)
**> 50**	41 (11.3%)
**Minutes allocated per outpatient**	SD = 12.2
**< 10**	233 (64.2%)
**10 ~ 20**	105 (28.9%)
**20 ~ 30**	23 (6.3%)
**> 30**	3 (0.8%)
**Inpatient practice**	*N* = 600 (%)
**No. of inpatients per day in the ward**	SD = 14.9
**≤ 5**	66 (11.0%)
**5 ~ 15**	363 (60.5%)
**15 ~ 25**	87 (14.5%)
**> 25**	84 (14.0%)

We also investigated the work status about outpatient and inpatient practices. As shown in Table 1, 363 respondents answered the outpatient practice. Among these respondents, they do outpatient clinic on average 2.65 times per week; they average see 28.8 outpatients per day; for every outpatient, they allocated about 12.2 min. 600 respondents answered the inpatient practice. They deal with average 14.9 inpatients per day in the ward. 60.5% of answered respondents deal with 5–15 inpatients per day, 84 respondents even need to deal with more than 25 inpatients per day. For most of the respondents didn’t answer the questions about outpatient and inpatient practice, the factors of work status were not included further analyzed.

Table 2 shows Burnout subscales’ scores according to demographic and work condition characteristics. The EE scores were significantly different among gender, age, working hour and professional department, the DP scores were significantly different among gender, age, education, working hours and professional department; the PA scores were significantly different among age, marital status, professional title and professional department. Higher mean scores of EE and DP were found among respondents who were male, 30–39 years old, had master’s degree or high and working hour > 60 h per week, came from obstetrics and gynecology or pediatrics profession. Lower mean scores of PA were found among respondents who were less than 30 years old, single and junior title, working in small specialist like ophthalmology and otorhinolaryngology, dermatology or physical examination.

**Table 2:** Burnout subscales’ scores according to personal and work characteristics

Factors	MBI-HSS					
	EE		DP		PA	
	Mean ± SD	***P value***	Mean ± SD	***P value***	Mean ± SD	***P value***
**Gender**		0.013		< 0.001		0.449
**Male**	25.30 ± 11.23		11.65 ± 6.68		30.76 ± 9.07	
**Female**	23.45 ± 10.78		9.72 ± 5.92		30.29 ± 9.09	
**Age(y)**^a^		0.001		0.035		0.005
**< 30**	22.18 ± 11.09		9.82 ± 6.09		28.51 ± 9.66	
**30–39**	25.10 ± 10.60		10.98 ± 6.30		30.46 ± 8.32	
**40–49**	24.94 ± 10.91		10.07 ± 6.22		31.49 ± 8.87	
**≥ 50**	21.48 ± 11.27		8.65 ± 5.67		32.70 ± 10.44	
**Marital status**		0.253		0.989		0.002
**Single**	23.17 ± 10.75		10.23 ± 5.95		28.82 ± 9.26	
**Married**	24.33 ± 10.99		10.29 ± 6.32		31.04 ± 8.91	
**Divorced or widowed**	22.25 ± 12.05		10.19 ± 5.90		30.50 ± 11.00	
**Education**^a^		0.149		0.002		0.069
**College degree or lower**	23.14 ± 10.99		9.65 ± 5.74		29.6 ± 9.72	
**Bachelor degree**	24.07 ± 11.28		10.02 ± 6.37		31.09 ± 9.17	
**Master ‘s degree or higher**	24.57 ± 10.20		11.35 ± 6.19		29.86 ± 8.21	
**Position**		0.213		0.032		0.001
**Doctor**	24.55 ± 10.72		10.78 ± 6.38		30.66 ± 8.51	
**Nurse**	23.17 ± 11.21		9.55 ± 5.84		29.98 ± 9.84	
**Professional Title**		0.241		0.714		0.036
**Junior**	23.72 ± 10.97		10.30 ± 6.17		29.70 ± 8.97	
**Middle**	23.75 ± 10.94		10.10 ± 6.36		31.11 ± 8.93	
**Senior**	25.20 ± 10.77		10.55 ± 6.01		31.17 ± 9.37	
**Income per month (CNY)**		0.171		0.124		0.449
**< 3000**	24.36 ± 11.51		10.08 ± 6.34		29.72 ± 9.62	
**3000-4999**	24.51 ± 10.66		10.67 ± 6.15		30.87 ± 8.68	
**5000-7999**	22.88 ± 10.76		9.75 ± 6.14		30.38 ± 9.25	
**> 8000**	24.90 ± 11.52		11.27 ± 6.36		29.83 ± 9.25	
**Working Hours Per Week (h)**^a^		< 0.001		< 0.001		0.857
**< 40**	19.52 ± 9.90		8.47 ± 5.92		30.60 ± 9.86	
**40–49**	22.84 ± 10.94		9.45 ± 6.09		30.34 ± 9.52	
**50–60**	25.39 ± 10.52		11.50 ± 6.33		30.51 ± 7.90	
**> 60**	28.01 ± 10.57		11.97 ± 5.94		30.56 ± 8.59	
**Professional department**		< 0.001		< 0.001		0.020
**Internal Medicine**	24.20 ± 11.24		10.00 ± 5.91		30.70 ± 9.24	
**Surgery/ Emergency/ ICU/Anesthesia or operating room**	24.53 ± 10.79		10.75 ± 6.50		30.26 ± 8.70	
**Obstetrics and Gynecology/ Pediatrics**	27.62 ± 11.38		12.29 ± 6.29		29.45 ± 9.17	
**Ophthalmology and Otorhinolaryngology/Dermatology/ physical examination**	22.44 ± 9.76		10.32 ± 5.19		28.38 ± 9.42	
**Auxiliary Clinical (Laboratory, Ultrasound, Pharmacy, Radiology)/others**	20.62 ± 10.00		10.28 ± 6.21		32.064 ± 9.08	

The difference was examine by Student’s *t* test and ANOVA. In the case of data with uneven variance, the Kruskal-Wallis nonparametric test was performed

^a^Perform Kruskal-Wallis test

**Fig. 1:**
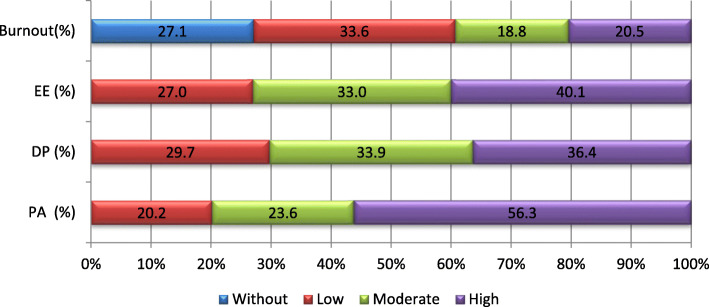
Prevalence of the burnout and its 3 subscales in the respondents

The prevalence of job burnout and its subscales was shown on Fig. 1. Among these medical staff, the prevalence of the high burnout is 20.5%, the moderate burnout is 18.8%, and the low burnout is 33.6%. Totally, 72.9% of all respondents reported a least one symptom of burnout. The prevalence of high level of each burnout subscale (EE, DP and PA) is 40.1, 36.4 and 56.3%, respectively.

**Fig. 2:**
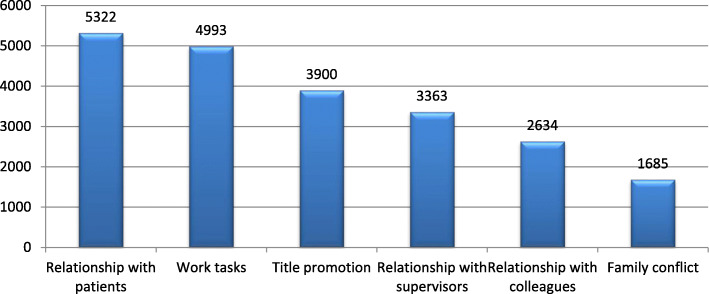
The rankings of six stressors among respondents

The ranking of subjective perception of six stressors among respondents was shown in Fig. 2. The ranking order is relationship with patients, work tasks, title promotion, relationship with supervisors, relationship with colleagues and work-family conflicts. The scores of these stressors in the ranking survey are 5322, 4993, 3900, 3363, 2634 and 1685, respectively.

Correlations between MBI-HSS subscales and WVS, FMS and ERI are detailed in Table 3. Both WVS and FMS showed positive correlations with EE and DP (*p* < 0.01), WVS showed negative correlation with PA (*p* < 0.01). For ERI subscales, both extrinsic effort and overcommitment showed positive with EE and DP (*p* < 0.01), extrinsic effort showed negative correlation with PA (*p* < 0.01). Reward had a negative with EE and DP, and a positive correlation with PA (*p* < 0.01).

**Table 3:** Correlations between MBI-HSS subscale scores and FMS, WVS, and ERI

Variables	Mean ± SD	MBI-HSS scores		
		EE	DP	PA
**MBI-HSS**				
**EE**	23.21 ± 10.94			
**DP**	10.27 ± 6.21	0.771^**^		
**PA**	30.42 ± 9.08	−0.099^**^	−0.231^**^	
**FMS**	20.96 ± 5.34	0.415^**^	0.323^**^	0.054
**WVS**	2.44 ± 3.29	0.296^**^	0.349^**^	−0.109^**^
**ERI**				
**Extrinsic effort**	19.44 ± 5.23	0.614**	0.478**	−0.047*
**Reward**	41.82 ± 9.05	−0.474**	−0.480**	0.219**
**Overcommitment**	16.37 ± 3.44	0.466**	0.332**	0.041

The Pearson correlation test were performed

*EE* Emotional exhaustion, *DP* Depersonalization, *PA* Personal accomplishment, *FMS* Fear of Malpractice Scale, *WVS* Workplace Violence Scale, *ERI* Effort-Reward Imbalance

**: *p* value < 0.01. *: *p* value < 0.05

The effect of different variables on the variance in burnout subscales scores were detailed in Table 4. Demographics variables explained 0.7% of variance in EE, 2.5% of variance in DP, and 1.6% of variance in PA. Work situations explained 5.6% of variance in EE, 2.7% of variance in DP, and 0.3% of variance in PA. The stressor of relationship with patients (WVS and FWS) was responsible for 19.2, 16.8 and 2.0% of variance in EE, DP and PA, respectively. The stressor of work tasks (ERI) were responsible for 23.5, 16.0 and 5.4% of variance in EE, DP and PA, respectively. Totally, these two stressor explained 42.7% variance in EE, 32.8% variance in DP and 7.4% variance in PA, respectively.

**Table 4:** The effects of different variables on the variance in MBI-HSS subscales scores

	EE	DP	PA
**Demographics**			
**F**	1.809	6.721**	4.221**
***R***^**2**^	0.007	0.025	0.016
**ΔR**^**2**^	0.007	0.025	0.016
**Work situations**			
**F**	12.387**	5.974**	0.614
***R***^**2**^	0.063	0.053	0.019
**ΔR**^**2**^	0.056	0.027	0.003
**The relationship with patients**			
**F**	132.770**	111.052**	10.519**
***R***^**2**^	0.255	0.221	0.039
**ΔR**^**2**^	0.192	0.168	0.020
**Work tasks**			
**F**	158.025**	88.763**	20.183**
***R***^**2**^	0.490	0.381	0.92
**ΔR**^**2**^	0.235	0.160	0.054

Demographics include age, gender, marital status, education; Work situations include title, income and working hour, professional department

Relationship with patients include WVS and FMS. Work tasks includes ERI

In step 1, demographics were added

In step 2, work situations were added

In step 3, the relationship patients were added

In step 4, work tasks was added

*MBI-HSS* Maslach Burnout Inventory-Human Service Survey, *EE* Emotional exhaustion, *DP* Depersonalization, *PA* Personal accomplishment, *ΔR*^*2*^ R^2^ increase, *FMS* Fear of Malpractice Scale, *WVS* Workplace Violence Scale, *ERI* Effort-Reward Imbalance

** *p* < 0.01

## Discussion

In this study, we performed a cross-sectional survey to investigate the prevalence and stressors of job burnout for medical staff in Liaoning, China. We reported 72.9% of respondents experienced at least one symptom of burnout. Respondents with high levels of EE, DP and low PA were 423 (40.1%), 384(36.4%) and 594 (56.3%), respectively. Our results were consistent with the previous studies for Chinese doctors and nurses [8, 9]. For the burnout definitions and criteria are diverse [4], it is difficult to compare burnout rates directly between studies. But it is clear that Chinese medical staff suffered with the high prevalence of burnout [9, 39–41]. Compared with a previous burnout study in Liaoning province in 2013 [15], our result of high burnout was much higher (20.5% verse 12.1%). The prevalence and situation of job burnout in Chinese medical staff is still serious [42, 43]. Hospital administrators should be aware about the risk of burnout and efforts should be made to reduce job burnout.

In this study, we noticed that the low PA (56.3%) was much higher than other survey for physicians in other countries [4]. Similarly high percent of low PA also was found in Chinese anesthesiologists (57%) [44], residents in standardized residency training (69.5%) [26] and registered nurses (93.5%) [9]. It was a common phenomenon in Chinese medical staff. It may due to the psychological gap between reward and social reputation. Chinese medical staff, especially in tertiary hospital, have relatively high social reputation. However, they have to suffer with long training period, work overload, medical responsibility, pressure for promotion and the tension relationship with patients but get relatively low income. There are universal dissatisfaction among Chinese medical staff. All these lead to reduce personal accomplishment of Chinese medical staff.

Job burnout is an individual experience among work context [2]. Job burnout can be effected with factors of demographics and work situations [44–47]. These demographics and work situations factors in our study explain variance in 6.3% EE, 5.2% variance in DP and 1.9% variance in PA. In our study, higher level burnout can be seen among respondents who were male, 30–39 years old, had master’s degree or high education, working hour > 60 h per week and from Obstetrics and Gynecology or Pediatrics department. It should be pay more attention on these medical staff for the psychological status.

Job stressors play an important role, especially for burnout outset [10]. Based on the notion that burnout is the end result of long exposure to chronic interpersonal stressors on the job, we selected six major stressors including work tasks, the relationship with patients, title promotion, the relationship with superiors, the relationship with colleagues, and work-family conflict for subjective perception survey. In another study for job burnout among Chinese healthcare professionals, they used an occupational stressors scale composed of 7 domains: organization and management, vocational interest, work load, career development, interpersonal relationship, external environment, and doctor–patient relationship [39]. The stressors we selected cover the similar content about the extrinsic perceived stressors in this scale. Our results showed that the relationship with patients and work tasks were the top two ranking stressors for Chinese medical staff, the stress from title promotion was another important stressor (Fig. 2). That is consistent with our expectation. These results reflected the attitude to different stressors among Chinese medical staff.

In this study, we used the ERI Questionnaire to evaluate the stress from work tasks. In our study, the ERI subscales had high correlations with EE and DP burnout subscales. The stress from work tasks (ERI) explained 23.5% of variance in EE, 16.0% variance in DP and 5.4% variance in PA, respectively. To assess the stress from the relationship with patient, we used the scales for workplace violence (WVS) and fear of malpractice (FMS). Our results indicated that both WVS and FMS were also significantly relative to the EE and DP subscales. The relationship with patient (WVS and FMS) explained 19.2% of variance in EE, 16.8% variance in DP and 2.0% variance in PA, respectively. It is interesting that both in the ranking survey and the regression analysis, these two stressors had roughly equivalent scores. Our results suggested that these two stressors both are the major indicators for job burnout.

The respondents we studied including doctors and nurses from different departments of tertiary general hospitals. Since different duties, there was part of them replied the questions about work status. The results showed that the medical staff in clinic need to see average 28.8 outpatients per day, most outpatients were allocated in less than 10 min. In the ward, every medical staff need to deal with average 14.9 inpatients per day. Except that, 88% respondents’ working hours exceed 40 h per week. These results reflected popular heavy burden among Chinese medical staff.

In China, due to the huge population and rapidly population aging, it creates increasing demands for medical care. From 2005 to 2015, the number of outpatient visits in China increased nearly four times, from 397 million to 1.5 billion per year [48, 49]. Most of Chinese medical staff experience overload, lack of hierarchical medical system aggravate this situation, especially in tertiary hospitals. Some doctors can see 70–80 patients in 1 day [50]. In our survey, 41 respondents reported they need to see more than 50 outpatients per day. The overload reduced the time allocated with per patient. Another study in a public tertiary general hospital in Southern China showed the average consultation time was about 5 min [49], compared with 10–20 min or more in Sweden and the United States [51]. Chinese medical staff have less time to communicate with patients. These combined effected strained the doctor(nurse)-patient relationship [15, 25], increasing workplace violence and popular fear of medical malpractice among medical staff. Meanwhile, the competition of title promotion in tertiary hospitals is very stressful. All these cause medical staff easy to exhaustion, thereby lead to job burnout.

Chinese policy-maker and hospital managers should pay more attention to the serious burnout status. There are some suggestions. The first is to ameliorate the work burden among medical staff, such as reducing working hours, caseloads, and on call periods. The overload is the pivotal factor related to burnout [2]. The hierarchical medical system should be established as soon as possible to reduce the pressure of tertiary hospitals. Another suggestion is to improve outpatient experience such as increasing consultation time and reducing waiting time, which is associated with patient satisfaction [49, 52]. Good food in the wards may be effective for inpatient satisfaction [53]. The improvement of patient experience is important for the relationship with patients. Psychosocial interventions such as relaxation or attention training maybe useful for managing occupational stress [54]. In tertiary hospitals, the incentive mechanism of title promotion should be reformed, research work should be included the routine job content, and allocate separate time. Except that, hospital managers should pay more attention to certain population such as more than 30 year old with junior title, etc. We also suggest to increase wages appropriately for medical staff of obstetrics and gynecology or pediatrics department etc.

At 2020, The outbreak and global of COVID-19 become a public health disaster. COVID-19 has become another stressor for medical staff. As our study is prior to the global pandemic, the impact of COVID-19 on burnout among Chinese medical staff should be discussed. From the reports of COVID-19 on burnout, the impact is diverse. It is reported that COVID-19 raised the high level of EE, but reduced low PA [55]. Another study showed that the medical staff working on the COVID-19 frontline ward had a lower frequency of burnout [56]. However, as burnout is the chronic response for stressors, COVID-19 will not immediately dramatically change the long term situation of burnout. Meanwhile, the stressors in our study still are the major stressors for Chinese medical staff, COVID-19 pandemic didn’t change it. COVID-19 outbreak will not change the conclusion of our study.

Several limitations must be mentioned in the present study. First, the participants in this study were limited to 8 tertiary hospitals from 3 cities in Liaoning province, the representation of study population may not be complete. Second, due to this study used a cross-sectional design, it was not able to determine causality relationships. Third, our study based on a self-reported questionnaire, the reporting variance may affect the results of burnout and other factors in our study. Fourth, the relationship with patient issue was measured using WVS and FMS, which are indirect scales. These factors should be considered in the in-depth studies.

## Conclusions

This survey for Chinese medical staff in Liaoning province found that the medical staff had high prevalence of burnout; 72.9% of them had at least one symptom of burnout and 20.5% experienced high burnout. The relationship with patients and work tasks are the major two stressors in respondent’s subjective perception survey. The stresses from the relationship with patients and work tasks are also the major indicators associated with job burnout. Our study underscore the need for hospital administrators to be aware of the risk of burnout. Efforts should be made to improve the relationship with patients and reduce stress from work, thereby ameliorating the status of job burnout.

## Data Availability

The datasets used and analyzed during the current study are available from the corresponding author on reasonable request.

## Abbreviations

EE: Emotional exhaustion
DP: Depersonalization
PA: Personal accomplishment
ERI: Effort-Reward Imbalance
WVS: Workplace Violence Scale
FMS: Fear of Malpractice Scale
